# Transcriptional and Genomic Targets of Neural Stem Cells for Functional Recovery after Hemorrhagic Stroke

**DOI:** 10.1155/2017/2412890

**Published:** 2017-01-04

**Authors:** Le Zhang, Wenjing Tao, Hua Feng, Yujie Chen

**Affiliations:** ^1^College of Computer and Information Science, Southwest University, Chongqing, China; ^2^Department of Neurosurgery, Southwest Hospital, Third Military Medical University, Chongqing, China

## Abstract

Hemorrhagic stroke is a life-threatening disease characterized by a sudden rupture of cerebral blood vessels, and it is widely believed that neural cell death occurs after exposure to blood metabolites or subsequently damaged cells. Neural stem cells (NSCs), which maintain neurogenesis and are found in subgranular zone and subventricular zone, are thought to be an endogenous neuroprotective mechanism for these brain injuries. However, due to the complexity of NSCs and their microenvironment, current strategies cannot satisfactorily enhance functional recovery after hemorrhagic stroke. It is well known that transcriptional and genomic pathways play important roles in ensuring the normal functions of NSCs, including proliferation, migration, differentiation, and neural reconnection. Recently, emerging evidence from the use of new technologies such as next-generation sequencing and transcriptome profiling has provided insight into our understanding of genomic function and regulation of NSCs. In the present article, we summarize and present the current data on the control of NSCs at both the transcriptional and genomic levels. Using bioinformatics methods, we sought to predict novel therapeutic targets of endogenous neurogenesis and exogenous NSC transplantation for functional recovery after hemorrhagic stroke, which could also advance our understanding of its pathophysiology.

## 1. Introduction

Hemorrhagic stroke, including intracerebral hemorrhage (ICH) and subarachnoid hemorrhage (SAH), is linked to high mortality and morbidity [1, 2]. Despite long-standing and worldwide efforts, the incidence of hemorrhagic stroke has not declined, according to a meta-analysis [3]. Currently, no effective medical treatment is available to improve the neurological outcomes in patients with hemorrhagic stroke. Although surgical decompression for cerebral hemorrhage benefits the survival of patients, defined pathogenesis and targets of prevention and treatment of hemorrhagic stroke have yet to be elucidated [4, 5]. Therefore, potential therapeutic strategies targeting secondary brain injury are attracting a lot of attention in translational studies of hemorrhagic stroke.

Neurogenesis is traditionally considered as an endogenous neuroprotective mechanism after acute central nervous system injuries, and it has been found to mainly occur in the subventricular zone (SVZ) along the lateral wall of the lateral ventricle and the subgranular zone (SGZ) of the dentate gyrus in the hippocampus [6]. Emerging evidence demonstrates that neurogenesis occurs after hemorrhagic stroke onset to repair the lesions of secondary brain injury and restore brain connections [7–9]. In addition, researchers have made great efforts to transplant exogenous neural stem cells (NSCs) to the brain lesions from different sources, including but not limited to embryonic stem cells, mesenchymal stem cells, and tissue-derived stem cells, with/without a variety of preinterventions. However, due to the complexity of the NSC microenvironment or niche, these strategies have either been proved unsatisfactory or resulted in serious side effects during clinical translation [10–12].

Recently, emerging evidence from the use of new technologies, such as next-generation sequencing and transcriptome profiling, has provided new insight into our understanding of genomic function and the regulation of NSCs. In this article, we will present current available data on controlling NSCs from both transcriptional and genomic levels. Using bioinformatics methods, we sought to summarize novel therapeutic strategies involving endogenous neurogenesis and exogenous NSC transplantation for functional recovery after hemorrhagic stroke, which could also advance understanding of the pathophysiology of hemorrhagic stroke.

## 2. Pathophysiology of Hemorrhagic Stroke

Primary brain injury after ICH happens in a few hours after the rupture of arteries resulting in bleeding and is mainly a result of hematoma formation with mechanical damage to adjacent tissues [1, 13]. For SAH, bleeding into the subarachnoid space due to aneurysm rupture leads to vasospasm and brain ischemia [14]. The hemorrhagic location and volume are highly associated with neurofunctional outcomes. However, the Surgical Trial in Intracerebral Hemorrhage (STICH trials I and II) has failed to provide convincing evidence to support the use of early surgical hematoma removal versus initial conservative therapy [15, 16]. In addition, recombinant activated factor VII significantly reduces hematoma growth without improving survival or functional outcomes in ICH patients (Clinical Trial: NCT00127283) [17]. Meanwhile, the treatment of SAH has not improved; the calcium channel blocker, nimodipine, is still the only proven drug to show beneficial outcomes for those patients with/without angiographic vasospasm. Additionally, treating vasospasm does not always lead to improvement in functional outcomes. This was recorded in randomized, placebo-controlled clinical trials (CONSCIOUS-2 and CONSCIOUS-3) using the endothelin receptor antagonist, Clazosentan, which reduced vasospasm in patients after SAH but failed to reduce mortality or attenuate neurological deficits.

Based on these disappointing results, researchers have turned their focus to mechanisms of secondary brain injury after hemorrhagic stroke, which play a critical role in the neurological deterioration in these patients [18–20]. Secondary damage is triggered from the blood components present that subsequently activate cytotoxic, excitotoxic, reactive oxygen species-related, and inflammatory-mediated pathways, and so forth. Nevertheless, neuroprotective agents, which have improved outcomes in animal studies, have failed to exhibit clinical benefits [21, 22]. Thus, strategies targeting NSCs and endogenous neurogenesis may be a potential and promising way to improve neurological outcomes after hemorrhagic stroke.

## 3. Current Understanding of the Neuroprotective Effects of NSCs for Hemorrhagic Stroke

### 3.1. NSCs for Hemorrhagic Stroke

The role of NSCs has been well defined in rodents, but neurogenesis in humans is more complicated. Histopathological examination of hippocampus tissue from cancer patients postmortem revealed the presence of nascent neurons [23, 24], providing the first evidence for human neurogenesis [23]. More recently, Spalding et al. retrospectively marked the hippocampal cells, by using the ratio of ^14^C to ^12^C in DNA of postmortem patients exposed to nuclear testing before death. Amazingly, they found that the turnover rate of new neurons in the dentate gyrus could be as high as 700 per day [25]. Meanwhile, by using two-photon laser scanning confocal microscopy, Shen et al. obtained specimens from patients with primary ICH and found that NSC specific proteins and cell proliferation markers were localized in cells in the perihematomal areas of basal ganglia and the parietal lobe after ICH [7]. These data suggest that ICH could induce de novo neurogenesis in the adult human brain. In addition, cerebral samples from SAH patients with aneurysm demonstrated the existence of many NSC markers, such as Nestin, vimentin, SOX-2, Musashi-1, and Musashi-2, which possibly contribute to the neural regeneration and functional recovery after aneurysm rupture [8]. However, elucidating the role of NSCs after hemorrhagic stroke in human still needs a large sample size of patients who vary in medical histories, cognitive ability, sportsmanship and lifestyles, and so forth, because all these factors can influence neurogenesis in experimental animals.

### 3.2. Neuroprotective Effects of NSCs after Hemorrhagic Stroke

Since the protective effects of neurogenesis are well reported in other acute central nervous system injuries, numerous researchers also support the beneficial role of NSCs after hemorrhagic stroke including proliferation, migration, and differentiation. Back in 2004, Tang et al. found that Nestin-stained or BrdU-labeled cells were mainly located in the basal ganglion and nearby SVZ around hematoma and ependyma after ICH in rats. Additionally, no cells positive for these markers were found in control or sham groups or in nonlesioned parenchyma [26]. Masuda et al. injected BrdU for two weeks after ICH in rats and found that BrdU-labeled cells significantly increased in both the contralateral and ipsilateral SVZs. Meanwhile, doublecortin-positive, immature, and migratory neurons were also seen in the dorsal striatum and perihematoma area two weeks after ICH. In addition, they also noticed clusters of doublecortin-stained cells in the striatum surrounding the hemorrhagic lesion four weeks after ICH. These findings implicate that experimental ICH induces the proliferation and migration of endogenous NSCs to repair the hemorrhagic lesion [9].

In addition to endogenous NSCs, exogenous NSC transplantation also exhibits the potential to attenuate neurological deficits after hemorrhagic stroke. In 2003, Jeong et al. intravenously transplanted human NSCs into experimental ICH rats. Their results indicated that NSCs can cross blood brain barrier and enter the rat brain with ICH. Interestingly, those surviving NSCs in the rat brain helped with the functional recovery [27]. Another investigation transplanted all-trans retinoic acid-induced NSCs into the contralateral ventricle up to 7 days after ICH and found new neurons and astrocytes surrounding the hematoma lesions of the brain four weeks later in all rats receiving the transplantations [28]. Moreover, these results were confirmed by superparamagnetic iron oxide- (SPIO-) labeled human NSCs detected by 3 T Magnetic Resonance Imaging, which indicated the presence of prominent NSCs in the periventricular region at four and six weeks after transplantation [29]. Most importantly, compared with the control group, the NSC-transplanted rats exhibited excellent functional performance on neurofunctional tests after two to eight weeks, which indicates that the exogenously supplied NSCs may be used for the functional recovery after hemorrhagic stroke [30].

### 3.3. Complexity of NSCs in Hemorrhagic Stroke Treatment

Despite the potential neuroprotective effects of NSCs, a lot of factors could influence the efficacy of NSC therapy for the hemorrhagic stroke treatment, such as intervention timepoint, administration routes, microenvironment of NSC, the source and status of NSCs, and possible immune responses.

According to a meta-analysis review, stem cell transplantation, particularly mesenchymal stem cell transplantation, significantly induces stem cell migration to lesion sites, decreases associated neural apoptosis and inflammation, improves ultrastructural integrity of cerebral tissue, and aids in improving neurologic function after SAH [31]. Additionally, intracerebral transplantation was the most effective route of administration for functional and structural recovery after ICH [32]. However, the effectiveness of the therapy in clinical practice remains to be determined [32].

Many factors such as metabolism regulators, epigenetic modifiers, vascular constrictors or dilators, modulators of immune response, and activators or inhibitors of signal transduction pathways can influence adult neurogenesis. Moreover, proliferation, differentiation, maintenance, and self-renewal of NSCs in the stem cell niche are controlled by a network of intrinsic and extrinsic regulators, such as neurotrophins, cyclins and cyclin-dependent kinases, and transcription factors. These factors act in concert within their biological network during the establishment and maintenance of neural connections. Epigenetic modulations during hippocampal development can also have impacts on one's learning and memorizing abilities. Genetic polymorphism in genes involving neurogenesis may have essential roles in variations of NSC differentiation between individuals in adult neural regeneration [33]. Elucidation of favorable genetic variations in neurogenesis may have therapeutic implications [33].

In mammals, new neurons are constantly generated in the SVZ and SGZ throughout developmental stage and adult life. This continuous neurogenesis after birth may be important in processing information, daily learning, memorization, and so forth. During hippocampal neurogenesis, doublecortin-positive immature neurons and neuronal precursor cells mature into neurons. In the immature stage, cells are sensitive and susceptible to extrinsic stimuli. However, knowledge on the dynamics which lead to neuron maturation is limited. Moreover, to date, purification of NSCs in vitro proves to be a challenging task to allow for investigation of their biology and application in clinical medicine.

By examining gene expression at single-cell level using RNA-seq technology, Gao et al. found that two subgroups existed among immature neurons with distinct gene expression profiles and different molecular markers. Comparisons of the two subgroups indicated that Notch and Sonic hedgehog (Shh) and the Hippo pathways are all important in neuron maturation and NSC activity [34, 35].

A complex network of elements, consisting of macromolecules of the extracellular matrix (ECM), support cells (glial cells/astrocytes/oligodendrocytes), adhesion molecules for cell-cell and cell-ECM connections, blood vessels, neurotrophins, and so forth, has an impact on tissue homeostasis and maintenance of a homing microenvironment for NSCs. Among these components, ECM derived from NSCs provides a unique and indispensable microenvironment that helps with stem cell differentiation and neural regeneration. Analysis of protein expression by two-dimensional gel electrophoresis and liquid chromatography-tandem mass spectrometry (LC-MS/MS) provided proteomic profiles that corresponded to unique niche properties for each group tested. Proteomic results demonstrated that NSC-derived ECM can impact the decision-making process of stem cell fate by offering microenvironment for specialized stem cell niches in the process of tissue development and regeneration [36].

## 4. New Insight into Genomic Function and Regulation of NSCs

Due to the development of omics (referring to the field of study in biology ending in -omics, such as genomics, proteomics, or metabolomics) technology, emerging evidence has demonstrated that both transcriptional and genomic pathways play important roles in ensuring the normal function of stem cells. At the transcriptional level, sequence-specific transcription factors and coregulators work together to orchestrate the transcriptional landscape of stem cells, which determines the on/off state of target genes, thereby controlling the cell fate of stem cells. At the genomic level, the replication and repair machineries maintain the genomic stability of stem cells.

The zebrafish is an excellent animal model because it can repair several organs, such as the damaged retina, severed spinal cord, injured brain and heart, and amputated fins. Recent technological developments of exquisite molecular tools for research in zebrafish, including cell ablation, lineage analysis, and novel and substantial microarrays, together with advancements in stem cell biology, have allowed scientists to investigate how progenitor cells contribute to the generation of appropriate structures and various underlying mechanisms, including reprogramming [37], and the appearance of various types of proliferating progenitor cell populations, such as SOX2^+^, A2B5^+^, and NG2^+^, of neural, glial/astrocyte, and oligodendrocyte progenitor cells, respectively. Among several essential factors for pluripotency, SOX2 and POU5F1 are significantly increased in neuron regeneration, which is linked to the pathway activation of progenitor cells. Elucidation of the fundamental mechanism for the endogenous neurogenesis and neuron network remodeling in adult zebrafish spinal cord has provided investigators with important ideas for future therapeutic strategies in acute brain injury repair and functional recovery in mammals [38]. Upon brain injury, neuronal progenitors of various types are recruited to the lesion site by different molecules. These progenitors are produced by the pool of NSCs to perform the task of regeneration. An imbalance of stem cell asymmetric division and self-renewal results in abnormal divisions and leads to the depletion of NSCs over time, which has been demonstrated in the alterations of the behavior of NSCs responsible for producing additional neurons in the process of neurogenesis [39].

Factors which form a regulatory network to support NSC self-renewal have not been fully elucidated up to now. Understanding of the key transcription factors (TF), the promoter region and other noncoding regions that they bind, and the target genes that they regulate, will be essential in unleashing the full potential of these cells for therapeutic use. At the center of this regulatory network are SOX family and FOX family TFs, nuclear factor I (NFI), and basic helix-loop-helix (bHLH) transcription factor family. Coordinated action of these factors to promote proliferation and at the same time prevent untimely differentiation and quiescence is crucial to NSC self-renewal [40]. By analyzing the region-specific regulatory networks based on available published databases on SVZ and SGZ, Ertaylan et al. discovered the potential microenvironment associated differences based on membrane and nuclear receptors via HIF-1*α*, Ar, and NR3C1. They also performed cell fate determinant test for NSCs from SVZ to the interneurons of olfactory bulb and NSC populations from SGZ to the granule cells of the granular cell layer. The existence of membrane and nuclear receptors in this region-specific regulatory network shows the importance of niche-derived extracellular molecules and region-specific factors for the neurogenesis in SGZ and SVZ [41].

Genomic approaches in modern time have facilitated unprecedented advances in our understanding of the development, function, and evolution of central nervous system. By contrast, little is recorded or published about the possible interplay between different genetic factors, epigenetic modulators, noncoding RNAs, and environmental factors in causing or modulating neurological disorders in populations from underdeveloped countries [42]. Both pharmacological intervention and genetic manipulation of epigenetic modulators can trigger profound changes in molecular expression, neuron identity, and complex behavioral and cognitive phenotypes. Apparently, epigenetics plays a nontrivial role in the pathogenesis of neurological disorders. Emerging paradigms in possible connections between epigenetics and hemorrhagic stroke include the following: how gene mutations of epigenetic factors induce hemorrhagic stroke; how genetic polymorphism of epigenetic factors is linked to disease risk of hemorrhagic stroke; how changes in the expression, localization, or function of epigenetic factors affect hemorrhagic stroke; how epigenetic factors modulate disease-linked genomic loci, protein expression, and cellular pathways; and how differential epigenetic profiles from patient-derived tissue samples affect disease outcome [43].

## 5. Bioinformatics Methods for Analyzing the Novel Therapeutic Targets of NSCs

Transcriptomic analysis, proteomic discovery, epigenetic status, and metabolic states during endogenous neurogenesis have the potential to lead to important discoveries and improve care of hemorrhagic stroke. Recent advances in analytic techniques present a new opportunity to discover potential targets that are of therapeutic values and provide new concepts which could change our perspectives of physiology, pathology, and biology in the near future.

Many research groups have studied the transcriptomics of NSCs and the process of NSC differentiation and cell fate determination to identify key regulators of NSC proliferation. Traditionally, Oct4 was found to be sufficient to reprogram human NSCs to pluripotency, with capacities for following proliferation and differentiation [44]. By doing transcriptome analysis at the single-cell level and weighted gene coexpression network analysis, Luo et al. were able to delineate the molecular characteristics of CD133^+^/GFAP^−^ ependymal cells from the forebrain neurogenic zone of adult mouse [45]. Single-cell sequencing has indicated that NSCs in many different activation states cooccur in the SVZ of adult brain [46] and that the network from adult NSCs forms a continuous linear trajectory [47]. Developmental genes such as Bcan, Fbln2, Itih3, Ncam1, Tnr, and Vcan modulate NSC differentiation via Wnt/*β*-catenin pathway at early stage of differentiation and TGF-*β* signaling pathway at later (7 day) stage. Of note, TGF-*β* pathway regulates epithelial to mesenchymal transition during development [48]. Transcriptome changes during the differentiation of human embryonic stem cells into neural lineage were identified to investigate the underlying mechanisms of neural differentiation [49]. TGIF1 and MARK1 have been found to be important during the development of cerebral cortex based on studies using human embryonic stem cells [50]. Moreover, Selective Reaction Monitoring-based proteomic profiling has allowed the creation of human pluripotent stem cell-derived neuronal model with reproducibility and physiological relevance. Combined with the quantification of proteins related to central nervous system diseases, this model provides the platform for potential drug discoveries [51].

Protein modifications posttranslationally are also a central part of NSC characterization which offer enormous information on such processes as cellular signaling, proliferation, differentiation, and maintenance. Studies based on expression profiles suggest that miRNAs are critical regulators in NSC biology [52]. Recently, neural stem cell maintenance was found to be regulated by an E2F1–miRNA feedback loop [53, 54]. A total of 10 miRNAs were identified to be differentially up- or downregulated in stem cells of glioblastoma versus normal NSCs, which may provide clues to develop miRNA-based therapies that target cancer stem cells specifically [55]. Recent studies have indicated key roles of miRNAs in reprogramming of somatic cells into NSCs or neurons [56–60]. In addition to miRNAs, transcripts over 200 nucleotides long which may not code for proteins and lncRNAs can have important biological functions in neuronal differentiation [61, 62]. Except for noncoding RNAs, other epigenetic mechanisms, such as DNA methylation and histone modifications, also play major roles in regulating and orchestrating gene expression during the course of neurogenesis as well as in neurological and psychiatric disorders [63–65]. The balanced DNA methylation status is essential for the maintenance and cell fate determination of neural stem cells during early development and in preventing malignant transformation [66, 67]. By using acetylated histone H3 ChIP-sequencing, the histone H3 acetylation level was found to increase overtime on the neural gene loci in the course of mouse embryonic stem cell differentiation to neurons, which revealed how the epigenetic modulation of histone acetylation/deacetylation coordinates with signals outside of the cells to determine the fate of NSCs [68]. However, our knowledge on the active roles of histone modifications in neurogenesis is only at the start line waiting to be developed [69].

The power of integrating different platform-based proteomics with the monitoring of multiple reactions was demonstrated [70], because small number of differentially expressed proteins did not show statistically significant differences in the outcomes of experimental group versus the control. A comprehensive review of NSC biology and epigenetics along with proteomics is beyond the scope of this manuscript [71]; we instead briefly summarize some basic information to show how proteomic technology has been widely used to indicate potential cellular targets mediating the differentiation of NSCs with regard to different aspects of multiple neurological diseases. Comparative proteomic analysis revealed HDGF as a novel angiogenic secreted factor during endogenous neurogenesis [72]. Membrane proteins expressed by the undifferentiated NSC line were identified [73].

A lot of work is now being devoted to developing innovative tools to ascertain the relationship between “omics” and analyzing the novel therapeutic targets of endogenous neurogenesis and exogenous NSC transplantation. For example, identification of cell fate determinants for directing stem cell differentiation remains a challenge. The gene-regulatory networks-based model of stem cell differentiation and computational method can guide differentiation experiments in stem cell biology and regenerative medicine [74]. However, the continuous development of computational and statistical methodologies will for sure provide greater precision and relevance of all “omics” research, without exceptions.

Additionally, identifying biomarkers of central nervous system disorders is one of the urgent goals of medicine in modern times. Most neurological disorders, including hemorrhagic stroke, are diagnosed too late due to the unavailability of biomarkers that can recognize early signs of pathological processes in the living brain. Like other omics fields, metabolomics may offer enormous information on the status of the brain at a given time point. By using proton magnetic resonance spectroscopy, Maletić-Savatić et al. discovered a metabolic biomarker of NSCs for the analysis in the live human brain, which connected systems with cellular neurobiology through the uses of certain specific metabolites. Thus, they give a functional observation into the living human brain, which may pave the way to the eventual discoveries of useful biomarkers of the diseases in clinic [75].

Reprogramming technology enables the production of NSCs from somatic cells by direct transdifferentiation. However, not much is recorded regarding how neuron processes in these NSCs or induced neural stem cells (iNSCs) behave differently from those of other stem cell populations both in vitro and in vivo. Hallmann et al. did transcriptome analyses on mouse iNSCs, which demonstrated unique, global, neural, metabolic, and cell cycle-related markers in these populations [76]. Xi et al. employed a mix of cytokines and small molecules to maintain the primitive and quiescent NSCs derived from mouse embryo stem cells and induced NSCs from rat fibroblasts by ectopic expression of three different transcriptional factors, including Oct4, Sox2, and c-Myc [77]. Clarification of the behavior of NSCs, in both clinical use and preclinical research, could predict well for the future brain tissue repair by transplantation of a patient's own-isolated stem cells [78]. However, poor reprogramming efficiency and the lack of proliferation of some somatic cell types make it hard to produce large numbers of neurons with this method and thus difficult to translate the technology into clinical use [79].

## 6. Perspective and Conclusion

**Figure 1:**
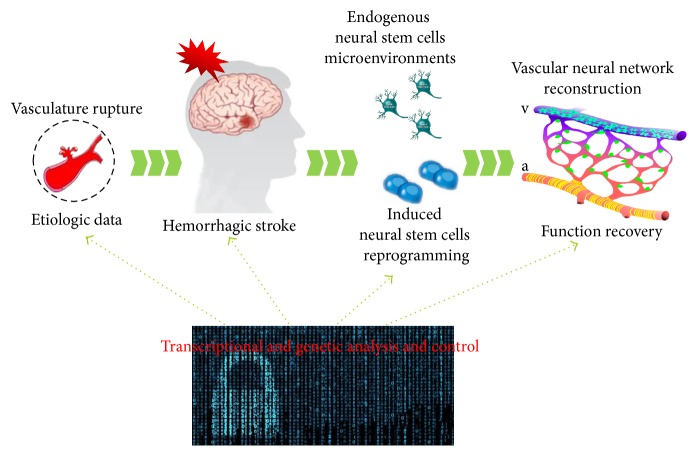
Diagram of transcriptional and genetic analysis and control for the function recovery in hemorrhagic stroke.

In the past twenty years or so, multiple technologies have been developed to utilize the regenerative potential of NSCs and the plasticity of neural cells in central nervous system to help preserve brain tissue after injury or improve structural and functional recovery upon acute brain injury, including hemorrhagic stroke [80]. Based on the pathophysiology of secondary brain injury after hemorrhagic stroke, targets regarding prediction, diagnosis, treatment strategies, and neurofunctional recovery need to be further identified and verified in large cohorts of patients, especially those controlling NSCs at both the transcription and genomic levels (Figure 1). Novel bioinformatics methods may provide much more information about therapeutic strategies for endogenous neurogenesis and exogenous NSC transplantation in hemorrhagic stroke management.
